# Appearance or attitude: what matters to craniosynostosis patients? Association of self-esteem, depressive symptoms, and facial aesthetics in patients with sagittal and metopic synostosis

**DOI:** 10.1007/s00381-022-05587-4

**Published:** 2022-06-23

**Authors:** Anja Svalina, Emma Heikura, Tuula Savolainen, Willy Serlo, Juha-Jaakko Sinikumpu, Anna-Sofia Silvola, Niina Salokorpi

**Affiliations:** 1 Research Unit of Clinical Neuroscience, Oulu University Hospital and University of Oulu, Oulu, Finland; 2 PEDEGO Research Group, University of Oulu, Oulu, Finland; 3 Medical Research Center, Oulu University Hospital, Oulu, Finland; 4 Department of Children and Adolescent, Oulu University Hospital, Oulu, Finland; 5 Department of Orthodontics, Faculty of Medicine, Oral Health Sciences, University of Oulu, Oulu, Finland; 6 Department of Neurosurgery, Oulu University Hospital, PO Box 21, 90029 Oulu, OYS Finland

**Keywords:** Metopic, Sagittal, Adult, Self-esteem, Depressive symptoms, Facial asymmetry

## Abstract

**Purpose:**

The aim of this study was to investigate the presence of depressive symptoms and self-esteem, and their association with facial asymmetry in adults who were operated on in early childhood due to metopic and sagittal craniosynostosis.

**Methods:**

The study population consisted of 49 non-syndromic patients of whom 41 had premature fusion of the sagittal and 8 of metopic suture. There were 64 controls from the Finnish National Register. Self-esteem, depressive symptoms, and subjective satisfaction with one’s appearance were evaluated by using the Rosenberg self-esteem questionnaire (RSE), the short form of Beck Depression Inventory (R-BDI), and a purpose-designed questionnaire on satisfaction with facial and overall appearance. Aesthetic evaluation was done from standard photographs using panels. The facial symmetry was calculated by using 3D photogrammetric methods.

**Results:**

Patients did not have a lower self-esteem or experience significantly more moderate or severe depressive symptoms. However, 20% of the patients and 6% of the controls (*p* = 0.041) experienced mild depressive symptoms. There was no difference between self-assessed evaluation of appearance between the groups. Only weak correlation was found between facial asymmetry and RSE or R-BDI results (cc = 0.27–0.42, *p* < 0.05). The self-assessed evaluation of facial and overall appearance correlated strongly with the R-BDI results.

**Conclusion:**

Non-syndromic craniosynostosis patients are as satisfied with their appearance in adulthood as the control group and do not experience a lower self-esteem or more depressive symptoms. Facial asymmetry does not correlate with low self-esteem or clinically significant depressive symptoms in adulthood. Subjective evaluation of one’s appearance correlated with depressive symptoms. Age and gender do not influence the former results. Overall, patients are satisfied with their appearance.

## Introduction

Craniosynostosis is defined as a condition where one or more cranial sutures are prematurely fused. It is the second most common congenital cause of infant deformity occurring roughly in 1 in 2000 live births [1, 2]. The premature fusion of the skull and facial bones may lead to aesthetical disturbances, malocclusion [3, 4], and psychological disorders [5, 6]. Abnormal head shape may develop depending on which sutures are prematurely fused, the order in which they ossify, and the timing at which it happens. The diagnosis is made by clinical examination along with additional imaging methods like computer tomography (CT) and magnetic resonance imaging (MRI) [7, 8].

An average human face is not fully symmetrical, and the amount of facial asymmetry in the normal population varies depending on the methodology used for evaluation [9]. Facial symmetry can be assessed by various methods, e.g., clinical evaluation, photography, cephalography, and three-dimensional (3D) imaging [9, 10]. Meta-analyses indicate that averageness, symmetry, and sexual dimorphism are all crucial parts of attractiveness in both male and female faces and across culture [11] with facial attractiveness being linked with various social advantages, fewer depressive symptoms, and greater self-esteem across the lifespan [12].

Craniosynostosis can influence facial attractiveness in adulthood through residual facial asymmetry, atypical head shape, and treatment-related scarring. Most facial symmetry analyses on craniosynostosis patients have been done on unilateral plagiocephaly and cleft or lip plate patients where the asymmetry has been found in mid or lower parts of the face [13–16]. In a recently published study, the forehead area was found to be the most asymmetrical area in scaphocephaly and trigonocephaly patients [17].

Craniosynostosis patients have been reported to have a less pleasing aesthetical appearance [18] and encounter social difficulties related to visible differences in appearance, such as teasing in childhood and difficulties in romantic relationships in adulthood [19, 20]. These factors have been found to predispose patients to experience lower self-esteem and worse psychosocial well-being in adulthood [21, 22]. However, the relationship between non-syndromic craniosynostosis, facial asymmetry, self-esteem, and depressive symptoms has been unclear.

Even though this condition has an enormous influence on the patient’s life, only a few studies have had long-term follow-ups through adolescence and adulthood [23]. Few studies have investigated how metopic or sagittal, non-syndromic craniosynostosis affects the aesthetics, self-esteem, and psychological well-being of adult patients [5, 19, 23–25].

Patients, who have suffered from deformities influencing their appearance since early childhood, could be expected to have a poor self-esteem and be prone to depressive symptoms. The present study aims to clarify is this valid for patients with treated scaphocephaly and trigonocephaly. Our goal was to investigate the presence of low self-esteem and depressive symptoms, and their correlation to facial characteristics and aesthetics of the patients operated on during early childhood because of sagittal or metopic craniosynostoses, compared to controls.

## Materials and methods

A total of 61 patients with isolated non-syndromic craniosynostoses who were treated in the Oulu University Hospital since 1977 and who were 18 years and older by December 2015 agreed to participate in the study. Data on long-term follow-up of the patients treated due to the sagittal suture synostosis and description of the study protocol itself has been published prior [26]. Patients who at the study visit appeared to have syndromic craniosynostosis (3), ventriculoperitoneal shunt (1), or plagiocephaly (8) were excluded from this study (*N* = 12). The final study group was comprised of 49 patients (32 males, 17 females), 41 of them were operated on due to scaphocephaly and 8 due to trigonocephaly. None of the patients had both scaphocephaly and trigonocephaly. Various operative techniques were used since the first operations were done in the late 1970s. For scaphocephaly patients, operative techniques included various forms of H-plasty with or without barrel stave osteotomies of the temporal bone technique for the operations (*N* = 25), the linear parasagittal craniotomy with silicone membrane interposition (*n* = 9), suturectomy together with dural split (*n* = 4), and suturectomy without dural split (*n* = 3). One trigonocephaly patient was operated using suturectomy with dural split; the rest of 7 cases underwent frontal cranioplasty using PDS (polydioxanone) sutures and metal wire; in one case, resorbable plates for fixation of the bone fragments were used. Patient’s mean age at operation was 5.6 months (ranging from 8 days to 45 months).

The results were analysed separately for scaphocephaly and trigonocephaly patients as well as for both diagnosis groups together. When analysing the patient groups separately, there was a statistically significant difference only in the trigonocephaly group (*N* = 8) when evaluating R-BDI according to subscores, which is unreliable due to statistical bias caused by very small population subgroups (5 trigonocephaly patients with R-BDI > 5 points, 3 patients < 5 points). Thus, the results of analyses done for the study population, including both scaphocephaly and trigonocephaly patients, are presented.

The control group consisted of age- and gender-matched participants, randomly chosen from a governmental database of the Finnish State Register. To provide a larger normal cohort, all 64 controls (32 males, 32 females) were included. For 10 controls, data on panel evaluation was not available; thus for the panel evaluation, 54 controls were used in this study.

### Evaluation of self-esteem and depressive symptoms

All patients and controls filled out the Rosenberg self-esteem (RSE) questionnaire, a widely used self-report measure on global self-esteem. The RSE consists of 10 questions with response options ranging from “strongly agree” to “strongly disagree”. The scale ranges from 0 to 30, with scores between 15 and 25 within normal range and scores below 15 considered to suggest low self-esteem [27].

Raitasalo’s modification of the short form Beck Depression Inventory (R-BDI), commonly utilized in the evaluation of mood in Finland, was used to evaluate depressive symptoms [28]. R-BDI consists of 13 questions with results ranging from 0 to 39 points. Scores below 5 are considered to indicate minimal depressive symptoms, scores between 5 − 7 mild depressive symptoms, 8 − 15 moderate depressive symptoms, and 16 or higher to indicate severe depressive symptoms [29].

### Evaluation of aesthetics

All patients and controls were administered a purpose-designed questionnaire regarding self-assessed satisfaction with facial and overall appearance in which the100-mm visual analogue scale (VAS) was used. In it, a response of 100 mm corresponded to “very satisfied” and 0 mm to “very unsatisfied”. This method and the results for soley scaphocephaly patients have been published previously [26].

Two different panels evaluated the aesthetic outcomes from photographs by using a 100 mm VAS scale with 0 mm as the least attractive and 100 mm being the most attractive. The first panel consisted of dentists specialised in orthodontics or orthognathic surgery, and the second panel consisted of five females and six males, all having no healthcare education. The method and results for the subgroup of soley scaphocephaly patients were described previously [26]. The dental panel and its evaluations for the subgroup of soley scaphocephaly patients were published previously [26].

### 3D analyses

The craniofacial characteristics were evaluated by using the 3dMDhead™ System (3dMD, Atlanta, GA, USA) with the previously described method [17] using the 21 Farkas landmarks and one additional landmark for the ear. The 3D images were processed and analysed using Rapidform 2006 (INUS Technology, Seoul, Korea). All landmarks were placed on the images by the first author. After that, all possible distinct parts (like hair and ears) were removed from each image in order to achieve the widest possible area of the face without disturbances for the analysis. Then, the position of the facial models was standardised using the previously described method [30]. In the lower face and chin measurements, we used 60 controls, excluding four controls due to facial hair in the chin area.

For measuring symmetry parameters, the facial surface was mirrored across the XY plane (mid-sagittal plane). Symmetry percentage (SP) was set to be the proportion of the facial areas where the distance between the original and the mirrored surface did not exceed 0.5 mm [17]. In this study, the SP was used for the whole face, forehead area, and chin area, and these sections were compared to the RSE and R-BDI results.

### Ethical board approval

The study was performed according to the principles of the Helsinki declaration. This is a part of a larger study approved by the Ethics Review Committee of the Northern Ostrobothnia Hospital District (No. 86/2013). Written informed consent was obtained from all participants.

### Statistical analyses

All statistical analyses were performed using the IBM SPSS software version 27 (SPSS Inc., Chicago, Illinois, USA). Two-tail *p* values of 0.05 or less were considered statistically significant. To assess normality, the Shapiro–Wilk normality test was used. Depending on the normality of the variables, the independent samples *T* test or Mann–Whitney *U* test was used. The Chi-squared test was used for nominal variables. Correlations between two variables were evaluated by Pearson or Spearman’s correlation depending on the normality of the variables.

## Results

The results of the symmetry percentages, RSE, R-BDI, and self-assessed satisfaction with facial and overall appearance were evaluated.

There was no statistically significant difference in experiencing depressive symptoms between the patient and control group (Table 1). The patients had a mean of 2.45 points, whereas the controls had 1.48 (*p* = 0.20). There were no differences between the males and females. Twenty per cent of the patients and 6% of the controls (*p* = 0.041) scored five or more points (indicative of mild depressive symptoms) on the R-BDI. However, if the limit was set for 8 or more points (moderate depressive symptoms), there was no statistical difference between the groups (8% vs 3%, *p* = 0.23).

**Table 1:** Self-esteem and depressive symptoms in the patient and control groups and their correlation with gender

	**Patients**				**Controls**				***p* value**
	Mean (SD)	95% CI	Median	IQR	Mean (SD)	95% CI	Median	IQR	
**R-BDI**	2.45 (4.20)	1.24–3.66	0.00		1.48 (3.67)	0.57–2.40	0.00		0.20^a^
Female	2.29 (4.14)	0.17.4.42	1.00		1.13 (1.90)	0.44–1.81	0.00		0.28^a^
Male	2.53 (4.30)	0.98–4.08	0.00		1.84 (4.83)	0.10–3.59	0.50		0.55^a^
5 ≥ points	20.4%				6.25%				0.041^c^*
8 ≥ points	8.2%				3.1%				0.23^c^
**RSE**	23.18 (6.26)	21.39–24.98	25.00		24.44 (5.07)	23.17–25.70	26.00		0.26^a^
Female	23.53 (5.82)	20.54–26.52	25.00	21.5–27.00	24.09 (4.99)	22.29–25.89	24.50	21.25–29.00	0.74^b^
Male	23.00 (6.57)	20.63–25.37	26.00	21.25–27.00	24.78 (5.20)	22.91–26.66	26.50	22.00–28.75	0.36^b^

*SD*, standard deviation; *IQR*, interquartile range, lower, and upper quartile; *95% CI*, 95% confidence interval. ^*^p value < 0.05

^a^Independent samples *T* test used

^b^Mann-Whitney *U* test used

^c^The Chi-squared test was used

There was no difference in self-assessed self-esteem scores between the study group and the control group (Table 1). Patients scored a mean of 23.18 points in the RSE score, whereas controls scored a mean of 24.44 points. Age did not correlate with the RSE or R-BDI results.

Both panels rated the patient group’s aesthetical appearance to be less pleasing than the control group’s (Table 2). The dentist panel median VAS was 6.24 mm for the patient group and 6.82 mm for the control group (*p* < 0.001). The lay panel median VAS was 4.95 mm and 6.17 mm, respectively (*p* < 0.001). Whether the scarring or the teeth were showing in the pictures did not have an impact on the results or correlations with the panels’ evaluation. There was no difference with self-evaluated VAS of own appearance between the two groups (Table 2).

**Table 2:** Subjective and objective VAS evaluations of aesthetics (mm)

	**Patients**				**Controls**				***p* value**
	Mean (SD)	95% CI	Median	IQR	Mean (SD)	95% CI	Median	IQR	
**Dentist panel**	5.97 (1.24)	5.61–6.32	6.24	4.88–6.93	6.82 (0.90)	6.58–7.07	6.82	6.28–7.48	< 0.001^b^*
**Lay panel**	4.99 (1.26)	4.63–5.36	4.95	3.94–5.93	6.07 (0.91)	5.82–6.31	6.17	5.34–6.74	< 0.001^b^*
**Q1**	7.21 (2.11)	6.61–7.82	7.80		7.51 (1.77)	7.07–7.95	7.80		0.42^a^
**Q2**	7.25 (2.12)	6.64–7.86	7.80		7.64 (1.66)	7.22–8.05	7.75		0.57^a^

*SD*, standard deviation; *IQR*, interquartile range, lower, and upper quartile; *95% CI*, 95% confidence interval. *p value < 0.05

*Q1*, self-assessed satisfaction with facial appearance; *Q2*, self-assessed satisfaction with general aesthetics

^a^Independent samples *T* test used

^b^Mann-Whitney *U* test used

The R-BDI scores correlated with the facial asymmetry estimates in all facial areas. In the patient group, the R-BDI results correlated weakly with the whole face and chin SP and in the control group weakly with the forehead SP*.* The satisfaction with appearance correlated with the R-BDI scores. A strong correlation was found between the R-BDI scores and satisfaction with facial appearance in the patient group (cc −0.51, *p* < 0.001) (cc = correlation coefficient). Additionally, a moderate to strong correlation was found between R-BDI and satisfaction with overall appearance (cc from 0.38 to 0.59, *p* < 0.001) in the patient group.

Self-assessed satisfaction with facial and overall appearance correlated strongly with the RSE score in both groups (Table 3) (cc 0.47–0.63, *p* < 0.01). In the patient group, weak correlation (cc 0.34, *p* = 0.020) was found between the RSE score and the whole face SP. Otherwise, facial symmetry did not correlate with the RSE results.

**Table 3:** Correlations between facial SP’s and self-assessed satisfaction with the RSE and R-BDI results, and the panels’ VAS evaluation of appearance

Symmetry parameter or question	RSE	R-BDI	Dentist panel	Lay panel
	Correlation coefficient (*p* value)			
**Whole face SP**				
Patients	0.34 (0.020)*	−0.35 (0.020)*	0.30 (0.048)*	0.29 (0.052)*
Controls	NS	NS	0.32 (0.010)*	NS
**Forehead SP**				
Patients	NS	NS	NS	NS
Controls	NS	−0.27 (0.033)*	0.36 (0.008)*	NS
**Chin SP**				
Patients	NS	−0.42 (0.005)*	NS	NS
Controls	NS	NS	NS	NS
**Q1**				
Patients	0.50 (< 0.001)*	−0.51 (< 0.001)*	NS	NS
Controls	0.47 (< 0.001)*	NS	NS	NS
**Q2**				
Patients	0.53 (< 0.001)*	−0.58 (< 0.001)*	NS	NS
Controls	0.63 (< 0.001)*	−0.39 (< 0.001)*	0.31 (0.02)*	0.28 (0.04)*

*Q1*, self-assessed satisfaction with facial appearance; *Q2*, self-assessed satisfaction with general aesthetics; *NS*, non-significant

^*^p value < 0.05

Dentist and lay panel’s evaluations on facial appearance correlated weakly or non-significantly in both groups with the whole face SP, forehead SP, and self-assessed satisfaction with facial appearance (Table 3).

## Discussion

This case–control study was conducted to evaluate the co-occurrence of low self-esteem and depressive symptoms with facial asymmetry and facial attractiveness in adults who were operated on in early childhood due to non-syndromic metopic and sagittal craniosynostosis. The central positive finding of the study was that satisfaction with appearance and self-esteem did not significantly differ between the patient and control groups. A small but significant difference was however found between groups in the occurrence of mild depressive symptoms. In addition, it was found that satisfaction with one’s own appearance in general as well as with facial appearance correlated with depressive symptoms. A weak correlation was observed between facial asymmetry and depressive symptoms as well as between the facial asymmetry and self-esteem in the patient group.

There was a statistically significant difference in panel assessment of facial attractiveness between patient and control groups, with the control group being rated more positively. This is in line with previous findings [18, 19] and likely attributable to differences in facial symmetry and head shape between groups, as smiling and the visibility of treatment scars had little impact on the panels’ evaluations (according to statistical calculations). However, the absolute differences in panel evaluations of appearance were relatively modest, especially in the case evaluations done by the dentist panel that were overall more positive with a smaller difference between groups and with most of the participants being rated at least average in appearance.

Previously mentioned findings are encouraging. This could be interpreted that while third parties may consider patients less attractive, patients themselves are satisfied with their own appearance. The majority of studies so far consider only the surgeon’s or parent’s opinions and do not consider the patient’s own opinions, the last being most important when taking in to account the overall well-being during the whole lifespan of the person.

Many studies have tried to find reasons why certain people experience more psychiatric symptoms and emotional problems than others [19, 31, 32]. In craniosynostosis patients, the degree of facial asymmetry and visibly different head shape has an observable effect on self-esteem and depressive symptoms [20, 31]. Noticeably, larger and more visible asymmetry (such as that in the forehead area) correlating with more significant psychological difficulties was observed [19, 33]. Facial asymmetry and attractiveness can impact self-esteem and depression through exposure to social disadvantages such as discrimination and by exposure to stigma and internalisation of stigma. These findings are thought to result from various psychological and social mechanisms. These mechanisms include having lower subjective satisfaction with one’s own facial appearance in childhood and adolescence [5, 6, 21], facing more social stigma and bullying in childhood and adolescence [19], and more difficulties in romantic relationships in adulthood [20]. Higher rates of negative social interaction are related to higher levels of depression and lower self-esteem in craniosynostosis patients in adolescence [20, 25]. However, visible facial differences may also counteract these negative effects by leading to increased resilience which in turn promotes better self-esteem and less depressive symptoms [20, 24].

In this study, the presence of depressive symptoms correlated strongly with lower satisfaction with one’s own appearance, weakly with asymmetry, and did not correlate at all with panel assessments. This is in line with previous findings [19]. Subjective satisfaction with appearance is more predictive of psychosocial distress than objective facial attractiveness [19], highlighting the importance of body positivity. Authors hypothesized that younger patients would have been more critical about their own appearance, and surprisingly, age and gender did not correlate with any evaluations. Summarizing these findings, the long follow-up results of operative treatment for non-syndromic metopic and sagittal craniosynostoses are good, leading to the same psychological status as in the age and gender verified population.

It is not clear what factors lead to a positive appearance in self-image development in craniosynostosis patients. Patient interviews and prior research [34] identify adolescence as a significant period regarding self-image development with positive and resilient outlooks:

“When I was in secondary school, the scar and different shape of the forehead bothered me but later on, I accepted my appearance and I’m satisfied with it.”

As absolute differences in symmetry analyses and panel evaluations between groups were small, and subjective evaluations of appearance were similar across groups, minor differences in self-esteem and depressive symptoms between groups were expected. Indeed, no observable difference in self-esteem measures was found. Depressive symptom scores thought to be indicative of mild depression in the R-BDI were more common in the patient group. Together with symmetry analyses, correlating with R-BDI scores, this presents weak support for the hypothesis that facial asymmetry can negatively influence the psychological well-being in adulthood. This is particularly significant, because to our knowledge, no prior studies have evaluated psychiatric symptoms or self-esteem measures of craniosynostosis patients in adulthood.

Future research should aim to identify both protective and risk factors regarding psychosocial outcomes of craniosynostosis patients in longitudinal settings where patients are followed from childhood to adulthood, taking into account family as well as cultural influences.

### Strengths and limitations

The strength and limitation of the study lies in analysing scaphocephaly and trigonocephaly patients as one group. In the authors’ opinion, this approach is well suited for the purposes of this study. Metopic and sagittal synostoses are the most common non-syndromic single suture synostoses, covering together over 80% of all craniosynostosis diagnoses. Although causing different types of dysmorphia, they are both caused by premature fusion of midline sutures and are not usually associated with asymmetry.

The strengths of this study included comparing the study population to a sizable control group, a long follow-up varying from 17 to 37 years after treatment, and the use of 3D soft tissue imaging, which is a modern and non-ionizing tool for analysing facial symmetry [1, 35, 36]. The BDI and RSE are most widely used instruments to evaluate depressive symptoms and self-esteem, and the Finnish versions have been found to be valid, repeatable, and reliable [27–29, 37].

The limitation is the subjective nature of evaluating aesthetics of the facial and overall appearance of one’s looks since evaluations can be affected by age, culture, and modern trends which change over time. Response style and subjective interpretation of questions may influence the questionnaire results. The presence of subtle learning difficulties or having a history of bullying were not controlled in this study. This is significant as non-syndromic craniosynostosis patients have been identified as having more subtle learning difficulties than reference groups [38, 39] and to experience more bullying, which could influence global self-esteem and depressive symptoms [5, 6, 21].

## Conclusion

Craniosynostosis patients do not experience significantly more moderate or severe depressive symptoms or lower self-esteem compared to controls in adulthood, nor does facial asymmetry influence them. Facial asymmetry did not correlate with the experiencing of depressive symptoms or low self-esteem. Age and gender did not correlate with any of the study parameters. Overall, craniosynostosis patients were satisfied with their appearance in adulthood.

## Data Availability

This manuscript has no associated data.
